# Comparative Histological and Histomorphometric Analysis of Xenografts and Synthetic Biomaterials in Rabbit Sinus Floor Elevation

**DOI:** 10.3390/jfb17090437

**Published:** 2026-09-01

**Authors:** Hirotomo Yanagiuchi, Daniele Botticelli, Erick Ricardo Silva, Samuel Porfirio Xavier, Giovanna Iezzi, Takahito Uwazumi, Shunsuke Baba

**Affiliations:** 1 Department of Oral Implantology, School of Dentistry, Osaka Dental University, Osaka-573-1121, Japan; yanagi-no-pc-yade@outlook.jp (H.Y.); uwazumi-t@cc.osaka-dent.ac.jp (T.U.); baba-s@cc.osaka-dent.ac.jp (S.B.); 2 Ardec Academy, 47921 Riccione, Italy; 3 Department of Oral and Maxillofacial Surgery and Periodontics, Faculty of Dentistry of Ribeirão Preto, University of São Paulo, Ribeirao Preto 14040-904, SP, Brazil; erick.silva@usp.br (E.R.S.); spx@forp.usp.br (S.P.X.); 4 Department of Medical, Oral and Biotechnological Sciences, University G. d’Annunzio of Chieti, 66100 Chieti, Italy; gio.iezzi@unich.it

**Keywords:** animal study, bone regeneration, bone substitutes, histomorphometry, interpenetrating bone network, maxillary sinus floor augmentation, Schneiderian membrane, synthetic biomaterials, xenogeneic bone substitute

## Abstract

Background: This study compared four biomaterials after maxillary sinus floor elevation in rabbits, focusing on graft persistence, tissue organization, space maintenance, and Schneiderian membrane response. Methods: Thirteen adult New Zealand White rabbits were enrolled, including 12 males and one female replacement animal; 12 animals provided evaluable specimens, with six assigned to each healing interval. Four elevated sinus compartments per evaluable animal were randomly allocated to Bio-Oss^®^, mp3^®^, ReproBone^®^ novo Paste, or Alos Granular. Specimens were collected after 2 or 10 weeks (*n* = 6 each). Histomorphometry quantified new bone, residual graft, soft tissue, elevated area, mucosal alterations, and IBN-like tissue, defined as areas in which biomaterial and bone could not be reliably separated. A combined graft and IBN percentage was calculated. Results: At 10 weeks, materials differed in residual graft (*p* = 0.0040) and combined graft and IBN percentage (*p* = 0.0218); Bio-Oss^®^ showed higher values than Alos Granular for both outcomes (adjusted *p* = 0.0024 and 0.0219, respectively). IBN-like tissue was higher in ReproBone^®^ novo Paste and Alos Granular than in Bio-Oss^®^ (adjusted *p* = 0.0219 and 0.0417), whereas only a small proportion occurred in mp3^®^. New bone did not differ among materials at either interval. Bio-Oss^®^ and mp3^®^ showed predominantly conventional osteoconduction. Alos Granular showed extensive degradation, whereas ReproBone^®^ novo Paste exhibited irregular graft and IBN regions. In the descriptive mucosal assessment, thinning was observed in all groups, whereas perforations were observed only at Bio-Oss^®^-treated sites; no inferential comparisons were performed for these outcomes. Conclusions: The materials differed in graft persistence, tissue architecture, dimensional stability, and mucosal response. Sinus graft evaluation should extend beyond new bone formation to include residual graft, IBN-like tissue, space maintenance, and mucosal compatibility.

## 1. Introduction

Posterior maxillary rehabilitation with dental implants may be limited by alveolar ridge resorption and sinus pneumatization, reducing the bone available for implant placement. Maxillary sinus floor elevation is therefore widely used to create a compartment in which bone regeneration can occur [1,2,3]. An ideal grafting material should support osteoconduction and bone formation while maintaining the elevated space and undergoing a biologically favorable remodeling process [4,5,6,7,8,9].

Deproteinized bovine bone mineral (DBBM), including Bio-Oss^®^, is among the most extensively investigated xenogeneic materials for sinus augmentation and is characterized by slow resorption and long-term persistence of residual particles [10,11,12,13,14,15,16,17,18,19]. Collagenated porcine xenografts such as OsteoBiol^®^ mp3^®^ have also been evaluated clinically and experimentally and show a more progressive remodeling pattern [20,21,22,23]. In contrast, synthetic substitutes avoid the use of animal-derived tissues [24,25] and may exhibit different degradation and tissue-integration characteristics depending on their composition, porosity, and microarchitecture [26,27,28,29,30,31,32].

The present study included two synthetic materials with markedly different characteristics. Alos Granular consists of porous non-sintered hydroxyapatite associated with a resorbable polylactic-co-glycolic acid copolymer, whereas ReproBone^®^ novo Paste is an injectable substitute based on nanocrystalline hydroxyapatite [33,34,35,36,37,38]. Evidence regarding their behavior in maxillary sinus floor elevation remains limited. Comparison with the more extensively investigated Bio-Oss^®^ and mp3^®^ therefore provides a reference for evaluating graft persistence, degradation, bone formation, and maintenance of the elevated compartment.

The time required for a grafting material to be replaced by newly formed bone cannot be defined as a uniform interval because it depends on material composition, microarchitecture, anatomical site, and local healing conditions. Bio-Oss^®^ undergoes very slow and incomplete resorption, with residual particles documented from 6 months to several years and even after 20 years [17,18]. Collagenated porcine xenografts such as mp3^®^ undergo progressive remodeling, although residual particles remain detectable in human sinus biopsies after approximately 6 months and during experimental healing periods [20,21,23]. For ReproBone^®^ novo Paste and Alos Granular, a material-specific time to complete replacement in the maxillary sinus has not been established; the available evidence describes scaffold degradation, bone ingrowth, and tissue integration rather than complete substitution within a predefined interval [33,34,35,36,37,38]. Accordingly, the 2- and 10-week intervals used in the present study represent early and later stages of remodeling and should not be interpreted as expected times of complete biomaterial replacement.

The biological response to a grafting material cannot always be described adequately by newly formed bone and conventional residual graft alone. Previous experimental observations with calcium phosphate biomaterials have identified an interpenetrating bone network (IBN), in which newly formed bone develops in close spatial association with remnants of degrading biomaterial rather than exclusively along its external surface [39,40]. Such patterns may influence the interpretation of graft persistence and tissue organization. In addition, graft characteristics may affect the overlying Schneiderian membrane, including mucosal thinning and perforation [41,42]. These tissue and mucosal responses may therefore provide complementary information on biomaterial performance.

Accordingly, the primary aim of the present study was to compare residual graft percentages among Bio-Oss^®^, mp3^®^, ReproBone^®^ novo Paste, and Alos Granular after maxillary sinus floor elevation in rabbits. Secondary outcomes included newly formed bone, soft tissue composition, dimensional maintenance of the elevated compartment, and Schneiderian membrane response. The occurrence and distribution of IBN-like tissue were additionally evaluated as exploratory histomorphometric outcomes.

## 2. Materials and Methods

### 2.1. Ethical Considerations

The experimental protocol received approval from the Animal Ethics Committee of the Faculty of Dentistry of Ribeirão Preto, University of São Paulo, Brazil, on 12 June 2024, under protocol number 0118/2024. Following the postoperative death of one animal, an amendment authorizing the enrollment of one additional rabbit was approved by the same committee on 14 August 2024 and assigned to the same experimental group and 10-week healing period to maintain the planned number of evaluable animals. All procedures were carried out in compliance with the regulations of the National Council for the Control of Animal Experimentation (CONCEA), and the study was reported following the ARRIVE 2.0 guidelines for animal research [43].

### 2.2. Research Design

Bilateral maxillary sinus augmentation was carried out in rabbits. In each sinus, two distinct access osteotomies were prepared, one in the anterior region and the other posteriorly. This design generated four elevated sinus regions per animal, which were randomly allocated to receive the four different biomaterials, without implant placement. All four biomaterials were placed during the same surgical session in each animal. Accordingly, the four experimental sites within a given animal underwent the same healing period, with six animals assigned to each of the 2- and 10-week intervals.

### 2.3. Animals and Eligibility Criteria

The study included adult male New Zealand White rabbits aged approximately 5–6 months and weighing 3.5–4.0 kg at the time of surgery. Following the postoperative death of one animal assigned to the 10-week healing interval, one adult female rabbit of similar age and weight was enrolled under an amendment approved by the Animal Ethics Committee. The animals were obtained from ANILAB (Paulínia, São Paulo, Brazil), a commercial breeding facility specializing in the supply of laboratory animals for scientific research, including New Zealand White rabbits. None of the animals had been used in previous experimental procedures.

Before enrollment, all rabbits underwent a veterinary examination and were confirmed to be clinically healthy. The inclusion criteria were an appropriate body weight, absence of systemic disease or infection, normal behavior, and suitability for general anesthesia and the planned surgical procedures. Exclusion criteria included any clinical abnormality that could interfere with surgery, postoperative recovery, animal welfare, or the study outcomes.

No animals were excluded following the preoperative veterinary examination.

### 2.4. Acclimatization, Housing, and Environmental Enrichment

Following arrival at the University of São Paulo, the rabbits were acclimatized to the institutional animal facility for at least 7 days before surgery.

Throughout the study, all animals were housed individually in the same animal room under identical environmental conditions. Stainless-steel cages providing a surface area of 4500 cm^2^ per animal were maintained under a 12 h light/dark cycle, controlled temperature (20–22 °C), and 15–25 air changes per hour. Standard laboratory chow and filtered water were available ad libitum.

Environmental enrichment consisted of a plastic resting platform provided in each cage throughout the experimental period.

### 2.5. Sample Size Calculation

The present investigation was designed as an exploratory preclinical study. Residual graft percentage at the final healing interval was selected as the primary quantitative endpoint. The sample-size calculation was based on the final 10-week measurements because different animals were evaluated at the 2- and 10-week healing intervals; therefore, individual longitudinal changes could not be calculated.

Previous rabbit maxillary sinus augmentation studies from our group reported residual graft percentages of 32.7 ± 7.6% for Bio-Oss^®^ [44] and 13.0 ± 5.4% for mp3^®^ at 10 weeks [23], corresponding to an expected mean difference of 19.7 percentage points. Because the present study used a within-animal design, the calculation was based on paired comparisons between each test material and Bio-Oss^®^. The intra-animal correlation between materials was not available from the previous studies and was conservatively assumed to be zero. This yielded an estimated standard deviation of the paired differences of 9.33 percentage points and an effect size d_z_ of 2.12.

The calculation was performed using G*Power, version 3.1.9.7 [45], adopting a two-sided matched-pairs t-test framework, a Bonferroni-adjusted α level of 0.0167 for the three predefined comparisons with Bio-Oss^®^, and a power of 80%. A minimum of six animals at the final healing interval was required. Accordingly, six animals were included at each healing interval.

Although the occurrence of IBN-like patterns was anticipated based on the composition and expected degradation behavior of Alos Granular and ReproBone^®^ novo Paste, as well as on previous observations with related synthetic calcium phosphate biomaterials, no quantitative data were available to estimate the expected amount, variability, or distribution of IBN-like tissue. IBN-like tissue was therefore not included in the sample-size calculation and was evaluated as an exploratory histomorphometric outcome.

### 2.6. Randomization, Allocation Concealment, and Blinding

Treatment allocation was generated electronically by an investigator (S.P.X.) who did not participate in animal selection, surgical interventions, or outcome evaluation. The anatomical position assigned to each biomaterial varied according to the randomization sequence. Within each animal, allocation was performed without replacement, so that each of the four biomaterials was assigned to one of the four anatomical sites and was used once per animal. The assigned biomaterial for each experimental site was recorded in opaque, sealed envelopes, which were opened only immediately before graft placement.

Histological specimens and slides were coded without explicit indication of the assigned biomaterial or healing interval. However, because the biomaterials exhibited distinctive histological morphologies, complete blinding to treatment allocation could not be guaranteed during microscopic evaluation.

### 2.7. Biomaterials

Bio-Oss^®^ spongiosa (granule size 0.25–1.0 mm; Geistlich Pharma AG, Wolhusen, Switzerland) is a bovine-derived deproteinized bone mineral characterized by a porous carbonate apatite structure [14].

OsteoBiol^®^ mp3^®^ (Tecnoss^®^, Giaveno, Italy) is a porcine-derived bone substitute composed of pre-hydrated collagenated cortico-cancellous bone granules, 600–1000 μm in size, mixed with collagen gel [21].

ReproBone^®^ novo Paste (Ceramisys Ltd., Sheffield, UK) is an injectable synthetic bone substitute based on an aqueous suspension of low-crystallinity nanocrystalline hydroxyapatite particles [38].

Alos Granular (Allmed S.r.l., Lissone, Italy), according to the manufacturer’s technical documentation, is a synthetic granular bone substitute composed of porous non-sintered hydroxyapatite associated with a resorbable polylactic-co-glycolic acid copolymer.

### 2.8. Anesthetic Protocol

Anesthesia was initiated by intramuscular administration of acepromazine at 1.0 mg/kg (Acepran^®^, Vetnil; Louveira, SP, Brazil), xylazine at 3.0 mg/kg (Dopaser^®^, Hertape Calier; Juatuba, MG, Brazil), and ketamine hydrochloride at 50.0 mg/kg (União Química Farmacêutica Nacional S/A; Embu-Guaçu, SP, Brazil). After sedation, the animals received prophylactic oxytetracycline IM at 0.2 mL/kg (Biovet; Vargem Grande Paulista, SP, Brazil), meloxicam 0.2% at 1.0 mg/kg SC (Flamavet^®^, União Química Farmacêutica Nacional S/A; Embu-Guaçu, SP, Brazil), and tramadol hydrochloride SC at 5.0 mg/kg (Halexistar; Goiânia, GO; Brazil).

Before surgery, the operative area was shaved and disinfected with a 1% iodine-based polyvinylpyrrolidone solution (Riodeíne^®^ Tincture, Rioquímica; São José do Rio Preto, SP, Brazil). Local anesthesia was then provided by infiltration of 2% mepivacaine with 1:100,000 norepinephrine (Mepinor^®^, Nova DFL; Rio de Janeiro, RJ, Brazil).

### 2.9. Surgical Procedures

All surgical procedures were carried out by a single highly experienced operator (E.R.S.). A midline incision approximately 2.0 cm in length was performed along the nasal dorsum. The underlying muscular tissues were gently dissected to expose the periosteum and the nasal bone.

**Figure 1:**
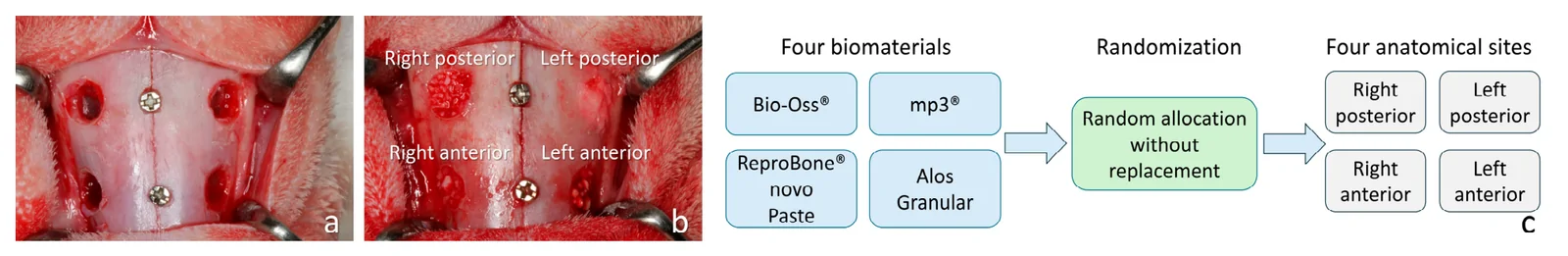
Surgical preparation and within-animal allocation of the biomaterials. (**a**) Two circular access windows were prepared on each side of the nasal dorsum, resulting in four experimental sites per animal. Small screws were placed along the naso-incisal suture at the level of the center of each osteotomy to serve as histological reference markers. (**b**) Representative intraoperative view after biomaterial placement, showing one example of the randomized allocation: Bio-Oss^®^ at the right posterior site, ReproBone^®^ novo Paste at the left posterior site, mp3^®^ at the right anterior site, and Alos Granular at the left anterior site. This material-to-site correspondence represents only the allocation used in the animal shown and was not fixed across animals. (**c**) Schematic representation of the within-animal allocation procedure. In each animal, Bio-Oss^®^, mp3^®^, ReproBone^®^ novo Paste, and Alos Granular were randomly allocated without replacement among the four anatomical sites, so that each site received one biomaterial and each biomaterial was used once per animal. The material-to-site correspondence varied among animals according to the randomization sequence; therefore, the schematic does not represent a fixed allocation.

On each side, an initial circular osteotomy measuring 5.5 mm in diameter was prepared with a trephine bur (S.I.N. Implant System, São Paulo, Brazil). This first window was positioned rostral to the nasofrontal suture and approximately 4 mm lateral to the naso-incisal suture. A second osteotomy was then prepared further rostrally, using the same approach. This resulted in two access windows per sinus and four experimental sites per animal. Small screws were placed in the naso-incisal suture at the level of the center of each osteotomy to serve as histological reference markers (Figure 1a).

After careful elevation of the sinus mucosa at all sites, the four elevated compartments were randomly allocated to receive one of the four biomaterials (Figure 1b). A nominal volume of 50 mm^3^ of each biomaterial was delivered using a 100-mm^3^ stainless-steel measuring spoon filled to half capacity (FK-05; Bontempi, San Giovanni in Marignano, RN, Italy).

At the end of the procedure, the periosteal layer was closed with resorbable sutures (Polyglactin 910 5-0, Vicryl^®^, Ethicon, Johnson & Johnson, São José dos Campos, Brazil), while skin closure was achieved using nylon sutures (Ethilon 4-0^®^, Ethicon).

### 2.10. Postoperative Care, Welfare Monitoring, and Humane Endpoints

Oxytetracycline was administered intramuscularly at a dose of 20 mg/kg. Postoperative analgesic and anti-inflammatory treatment consisted of subcutaneous meloxicam at 0.5 mg/kg once daily for 3 days (Flamavet, União Química Farmacêutica S/A) and subcutaneous tramadol hydrochloride at 5.0 mg/kg once daily for 3 days (Halexistar).

Animals were monitored daily by the research team, the attending veterinarian, and the animal facility staff. Clinical evaluation included food and water intake, urinary and fecal output, behavior, signs of pain or distress, general health status, and surgical wound healing, including swelling, bleeding, dehiscence, and evidence of infection.

Predefined humane endpoints included persistent anorexia or dehydration, uncontrolled pain despite analgesic treatment, severe infection, wound dehiscence compromising healing, marked impairment of mobility, progressive deterioration of general health, or any condition considered incompatible with continuation in the study. Animals meeting any of these criteria were to be immediately evaluated by the attending veterinarian and, when recovery was considered unlikely, humanely euthanized according to institutional guidelines.

### 2.11. Euthanasia

Animals were euthanized at the predefined healing intervals of 2 and 10 weeks, according to the experimental allocation. Before euthanasia, sedation was induced by intramuscular administration of acepromazine at 1.0 mg/kg (Acepran^®^, Vetnil), xylazine at 3.0 mg/kg (Dopaser^®^, Hertape Calier), and ketamine hydrochloride at 50 mg/kg (Ketamin Agener, União Química Farmacêutica S/A).

Once adequate sedation had been achieved, the animals were transferred to a carbon dioxide chamber. CO_2_ was delivered at a controlled flow rate of 7 L/min, corresponding to approximately 20% of the chamber volume. Gas administration was maintained for at least one additional minute after clinical death had been confirmed. Death was verified by the absence of respiratory movements, mucosal cyanosis, and lack of palpable pulse.

### 2.12. Histological Processing

The harvested specimens were dehydrated over six days in a graded ethanol series, progressing from 50% to absolute ethanol. They were then progressively infiltrated with increasing concentrations of resin, from 50% to 100% LR White™ HardGrid (London Resin Co., Ltd., Berkshire, UK). Resin polymerization was completed in an oven at 60 °C for 24 h.

Following polymerization, the blocks were sectioned longitudinally in the bucco-lingual plane using a precision diamond disk. The small screws previously positioned along the naso-incisal suture were used as reference landmarks to guide the cutting plane through the center of the corresponding osteotomies. Sections were initially obtained at a thickness of approximately 150 µm and subsequently reduced to about 30 µm using a dedicated grinding system. The resulting histological sections were then stained with acid fuchsin-toluidine blue.

### 2.13. Histomorphometric Outcomes and Measurements

Histological evaluation was performed by a calibrated examiner (E.F.D.R.) under the supervision of an experienced histologist (D.B.). Intra-examiner agreement for tissue classification, assessed by repeated evaluation of selected histological fields, yielded a kappa coefficient >0.90. Sections were examined using an Eclipse 50i light microscope equipped with a Digital Sight DS-Fi2 camera and connected to a computer-based image analysis system (Nikon Corporation, Tokyo, Japan).

**Figure 2:**
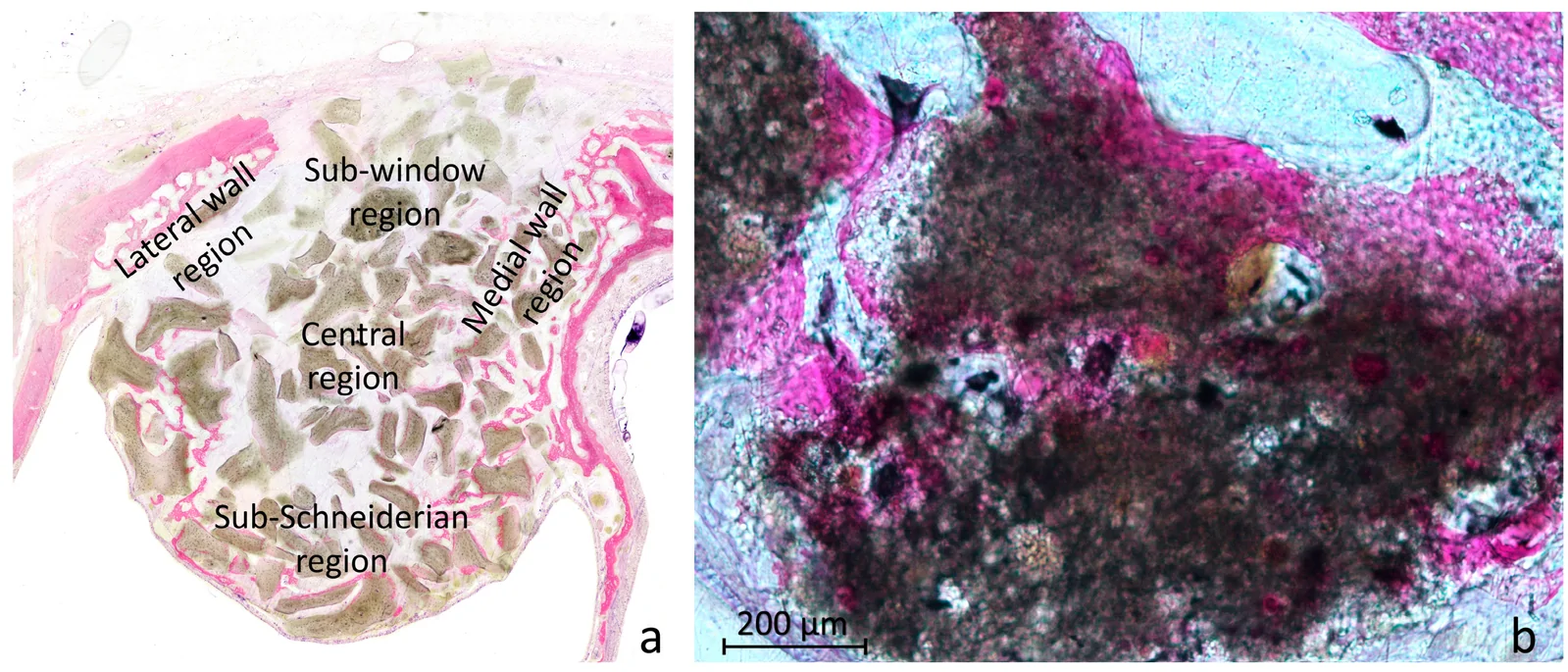
Histomorphometric sampling and identification of IBN-like tissue. (**a**) Representative histological section showing the regions systematically sampled for point-counting analysis: sub-Schneiderian, medial and lateral wall, central, and sub-window regions. One or two non-overlapping fields were analyzed in the sub-Schneiderian region depending on the extension of the elevated compartment. (**b**) Representative ReproBone^®^ novo Paste specimen after 10 weeks showing an IBN-like area. Increased transmitted-light intensity was used to facilitate visualization of newly formed bone closely intermingled with or optically superimposed on residual biomaterial, preventing unequivocal classification of these areas as either bone or graft. Acid fuchsin–toluidine blue stain. Scale bar = 200 μm.

Histomorphometric measurements were performed using a point-counting method. Sections were examined with a 10× objective, and a lattice grid with 75 µm squares, covering approximately 1200 × 900 µm, was superimposed on the digital microscope image. The grid comprised 16 columns and 12 rows of squares, corresponding to 17 × 13 grid-line intersections. All intersections, including those along the grid perimeter, were used as counting points, yielding 221 points per field. The following regions were systematically sampled: the sub-Schneiderian region, the medial and lateral wall regions, the central region, and the sub-window region (Figure 2a).

One field was evaluated in each of the medial wall, lateral wall, central, and sub-window regions, together with one or two non-overlapping fields in the sub-Schneiderian region, depending on the extension of the elevated compartment. Thus, five or six fields, corresponding to 1105 or 1326 counting points, respectively, were analyzed per experimental site. Counts from all sampled fields were pooled to obtain a single overall value for each tissue category at each experimental site. Regional measurements of newly formed bone were used only to describe its spatial distribution within the elevated compartment.

Four mutually exclusive tissue categories were recorded: newly formed bone, graft-only tissue, soft tissue, and interpenetrating bone network-like (IBN-like) tissue. Graft-only points were those containing identifiable residual biomaterial without newly formed bone. A point was classified as IBN-like when residual biomaterial and newly formed bone were closely intermingled or optically superimposed and could not be unequivocally assigned to either category. In these areas, increasing the transmitted-light intensity and adjusting the focal plane facilitated visualization of newly formed bone within, behind, or in apparent continuity with the residual biomaterial (Figure 2b).

For each experimental site, the percentage of each tissue category was calculated relative to the total number of evaluated points. Residual graft percentage, corresponding to the graft-only category, was designated as the primary histomorphometric outcome. IBN-like tissue was analyzed separately as an exploratory outcome. Because residual biomaterial was present in both graft-only and IBN-like points, a combined graft and IBN percentage was also calculated as the sum of these two categories to estimate the proportion of the augmented compartment containing residual biomaterial. IBN-like points were not added to the newly formed bone percentage because the relative proportions of bone and biomaterial within the same counting point could not be reliably quantified.

The Schneiderian membrane was assessed for thickness, focal thinning, and perforations. Mucosal thinning was defined as a thickness <40 μm [41,42]. For each specimen, the number and thickness of thinned sites were recorded. A perforation was defined as focal interruption of the epithelial lining with loss of normal mucosal continuity; processing-related tears or discontinuities were excluded.

Finally, the cross-sectional area of the elevated compartment was measured on each histological section and expressed in mm^2^. This two-dimensional measurement was used as a descriptive indicator of compartment dimensions at each healing interval and was not considered a direct measurement of augmented volume.

### 2.14. Statistical Analysis

The experimental design included two factors: biomaterial and healing interval. Biomaterial comprised four levels (Bio-Oss^®^, mp3^®^, ReproBone^®^ novo Paste, and Alos Granular) and was treated as a within-animal factor because all four biomaterials were evaluated in each animal. Healing interval comprised two levels (2 and 10 weeks) and was treated as a between-animal factor because different animals were assigned to each interval. Biomaterial comparisons were therefore performed separately at 2 and 10 weeks, and no inferential comparisons between healing intervals were planned.

Residual graft percentage (graft-only tissue) was the primary quantitative endpoint. Newly formed bone was considered a secondary endpoint, whereas IBN-like tissue, soft tissue, and combined graft and IBN were considered exploratory outcomes.

Normality of the overall histomorphometric data was assessed using the Shapiro–Wilk test, and homogeneity of variances was evaluated using the Brown–Forsythe test. Because of the small sample size and because the results of these assumption checks did not consistently support parametric assumptions, non-parametric methods were used for inferential analyses.

At each healing interval, differences among the four biomaterials were assessed using the Friedman test, with the animal as the experimental unit and the four biomaterial-treated sites as matched observations. When the Friedman test was significant, predefined comparisons between Bio-Oss^®^ and each test material were performed using Dunn’s multiple-comparison test, and adjusted *p*-values were reported.

Schneiderian membrane-related variables were analyzed descriptively, and no inferential comparisons among biomaterials or between healing intervals were performed for mucosal thinning or perforations. Cross-sectional area of the elevated compartment and regional histomorphometric measurements were likewise analyzed descriptively. Complete matched observations from six animals were available at each healing interval. Data are presented as mean ± standard deviation. Statistical analyses were performed using GraphPad Prism, version 11.0 (GraphPad Software, Boston, MA, USA). All tests were two-sided, and *p* < 0.05 was considered statistically significant.

## 3. Results

### 3.1. Animal Flow and Adverse Events

One rabbit assigned to the 10-week healing period developed a postoperative infection and died during the experimental period. No samples or data were obtained from this animal. One additional rabbit was enrolled under an amendment approved by the Animal Ethics Committee and assigned to the same healing period to maintain six evaluable animals. No further postoperative complications or unexpected adverse events occurred.

### 3.2. Qualitative Morphologic Evaluation

The four biomaterials displayed different healing patterns at both observation periods.

**Figure 3:**
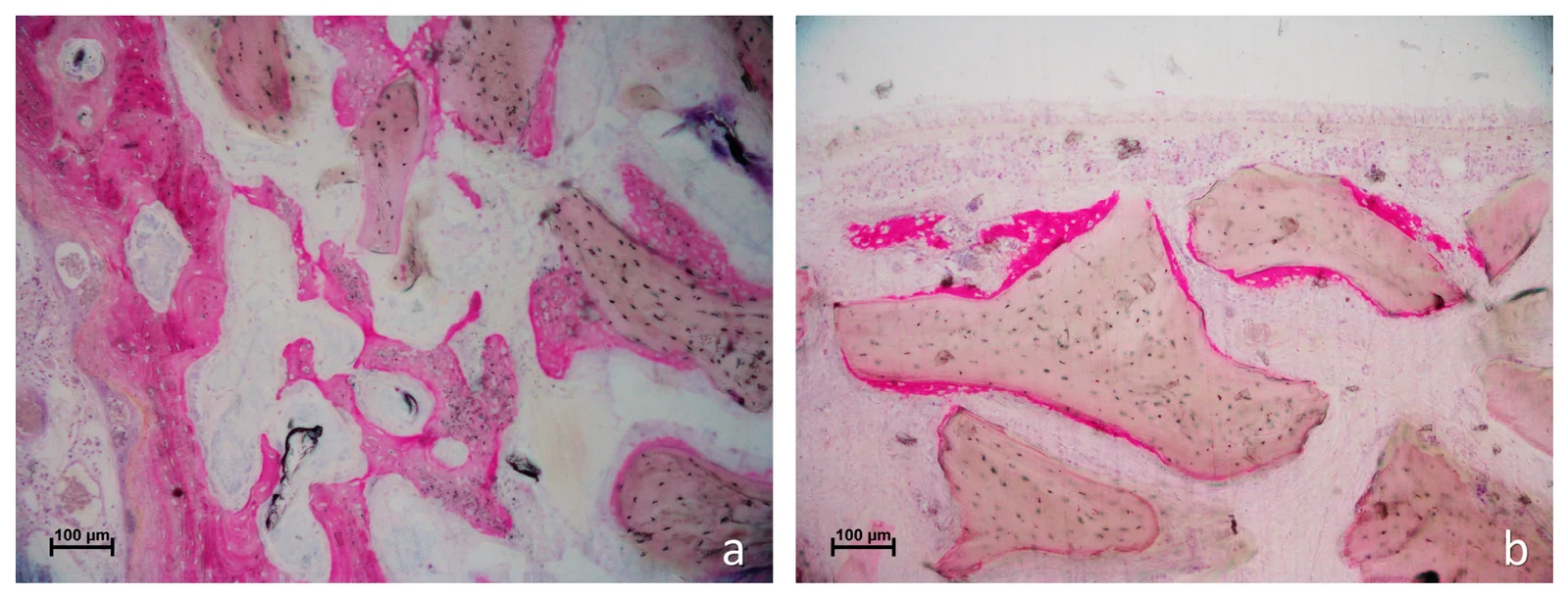
Representative histological images of the Bio-Oss^®^ group after 2 weeks of healing. (**a**) Newly formed bone was present throughout the elevated compartment but was more evident close to the native sinus walls, where thin trabeculae extended from the parent bone and partially lined the surfaces of residual Bio-Oss^®^ granules. (**b**) In the sub-Schneiderian region, newly formed bone was also observed close to the sinus mucosa, mainly along the surface of the granules and, in some areas, interposed between the biomaterial and the mucosal layer. Acid fuchsin-toluidine blue stain. Scale bars = 100 μm.

In the Bio-Oss^®^ group, newly formed bone at 2 weeks consisted mainly of thin trabeculae extending from the native bone walls and along the surfaces of residual granules (Figure 3).

**Figure 4:**
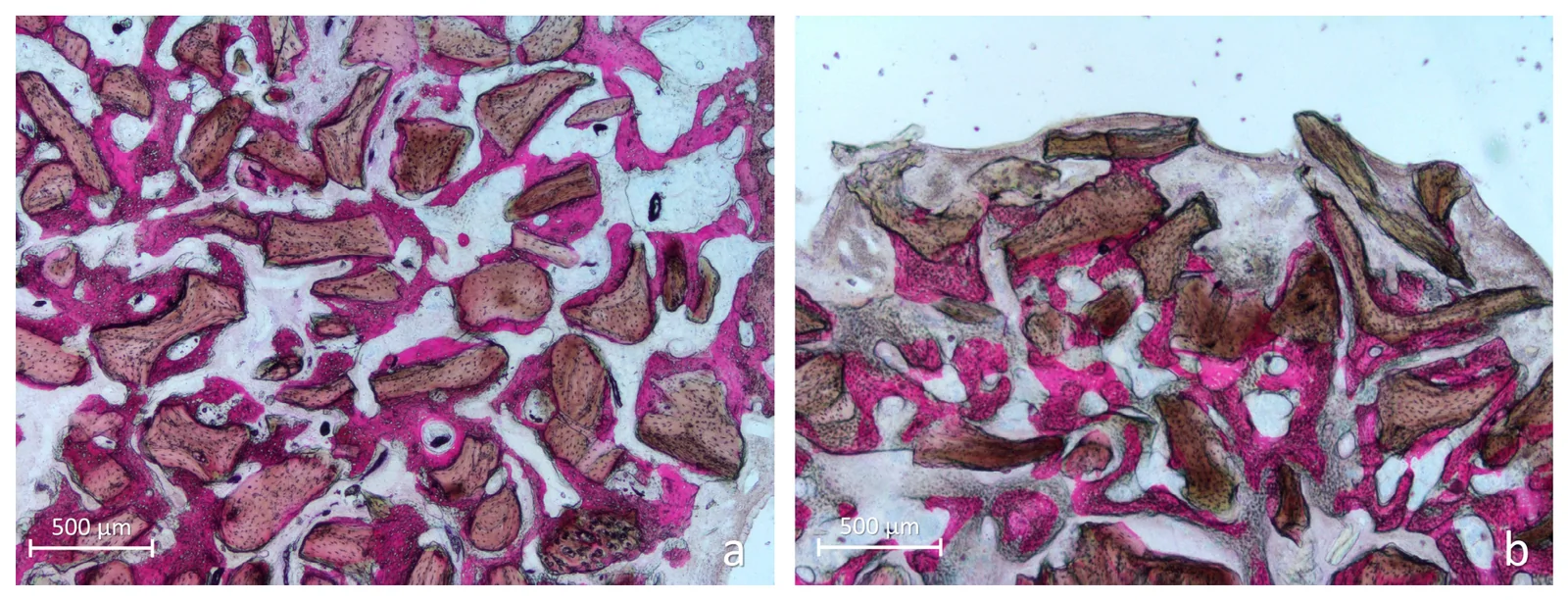
Representative histological images of the Bio-Oss^®^ group after 10 weeks of healing. (**a**) Newly formed bone was widely distributed throughout the elevated compartment and frequently surrounded or bridged adjacent residual Bio-Oss^®^ granules, progressively incorporating them within the mineralized trabecular network. (**b**) In the sub-Schneiderian region, the mucosal-facing surfaces of some granules remained devoid of newly formed bone and were associated with focal thinning and perforation of the sinus mucosa. Acid fuchsin-toluidine blue stain. Scale bars = 500 μm.

At 10 weeks, bone was more widely distributed and frequently surrounded or bridged adjacent particles (Figure 4a). In the sub-Schneiderian region, some mucosal-facing granules were associated with thinning or perforation of the sinus mucosa (Figure 4b).

**Figure 5:**
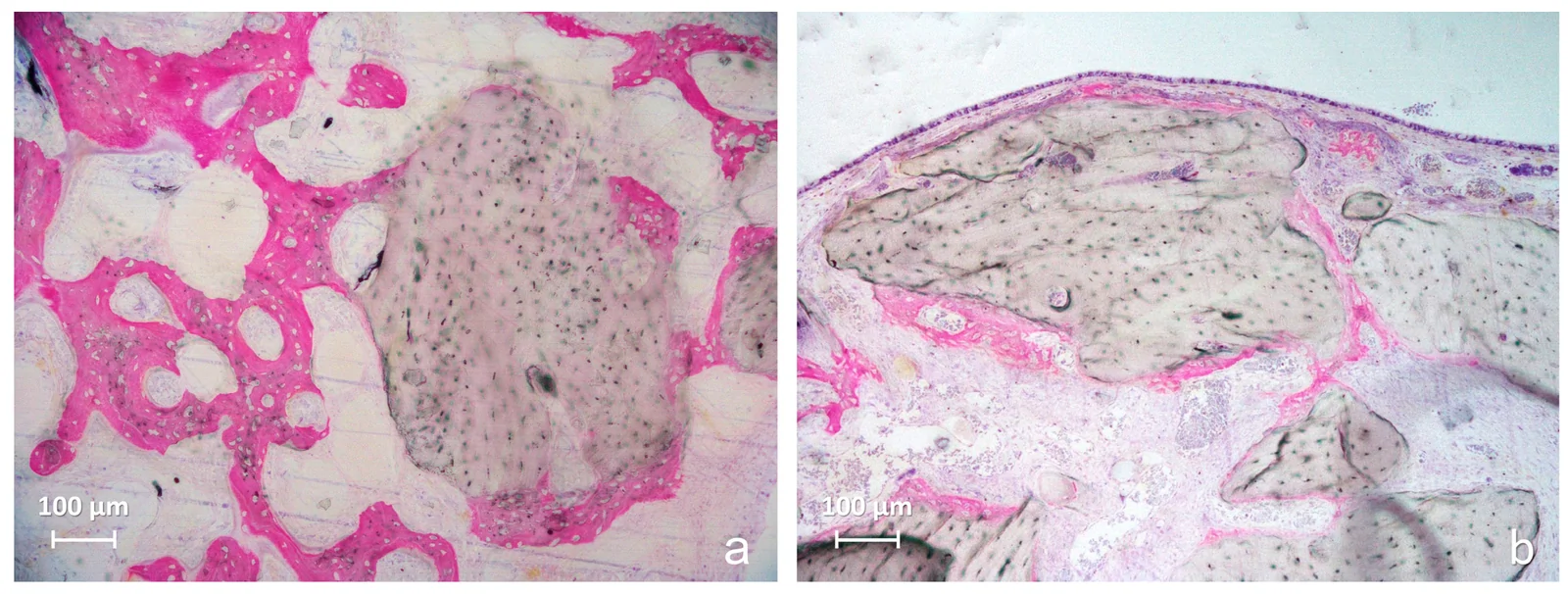
Representative histological images of the mp3^®^ group after 2 weeks of healing. (**a**) Newly formed bone was observed mainly in continuity with the native sinus walls, extending toward the elevated compartment and contacting residual mp3^®^ granules. (**b**) In the sub-Schneiderian region, newly formed bone was occasionally present close to the sinus mucosa, either along the surface of residual granules or interposed between the biomaterial and the mucosal layer. Acid fuchsin-toluidine blue stain. Scale bars = 100 μm.

In the mp3^®^ group, bone formation at 2 weeks occurred predominantly in continuity with the sinus bone walls, with occasional bone formation near the sinus mucosa (Figure 5).

**Figure 6:**
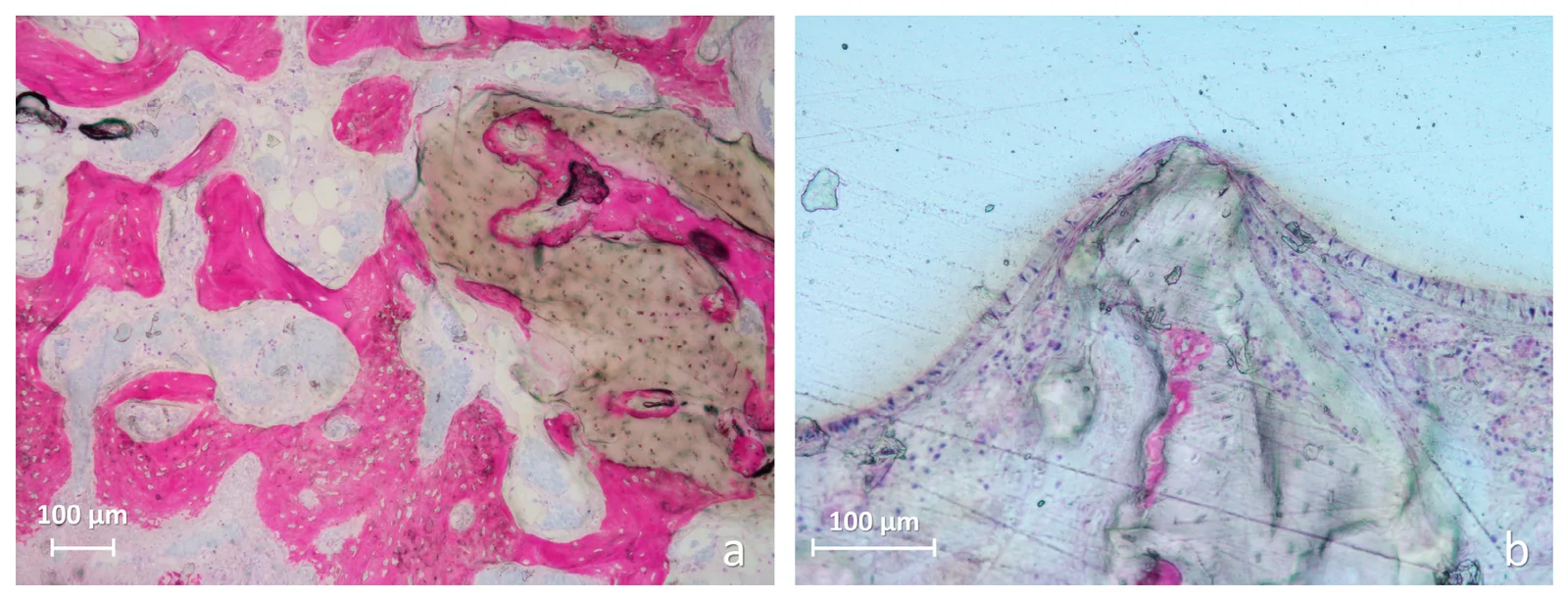
Representative histological images of the mp3^®^ group after 10 weeks of healing. (**a**) Residual mp3^®^ granules were still present and were frequently surrounded by newly formed bone and incorporated within the mineralized trabecular network. A small focal IBN-like area was identified in one specimen, although the overall healing pattern remained predominantly surface-related. (**b**) In the sub-Schneiderian region, newly formed bone showed areas with a more cortical-like organization. Residual granules located close to the sinus mucosa were associated with focal mucosal thinning, whereas no perforations were observed. Acid fuchsin-toluidine blue stain. Scale bars = 100 μm.

At 10 weeks, residual granules were frequently surrounded by newly formed bone, and the overall architecture remained predominantly trabecular (Figure 6a). A small focal IBN-like area was observed in one specimen. Localized mucosal thinning was present, but no perforations were detected (Figure 6b).

**Figure 7:**
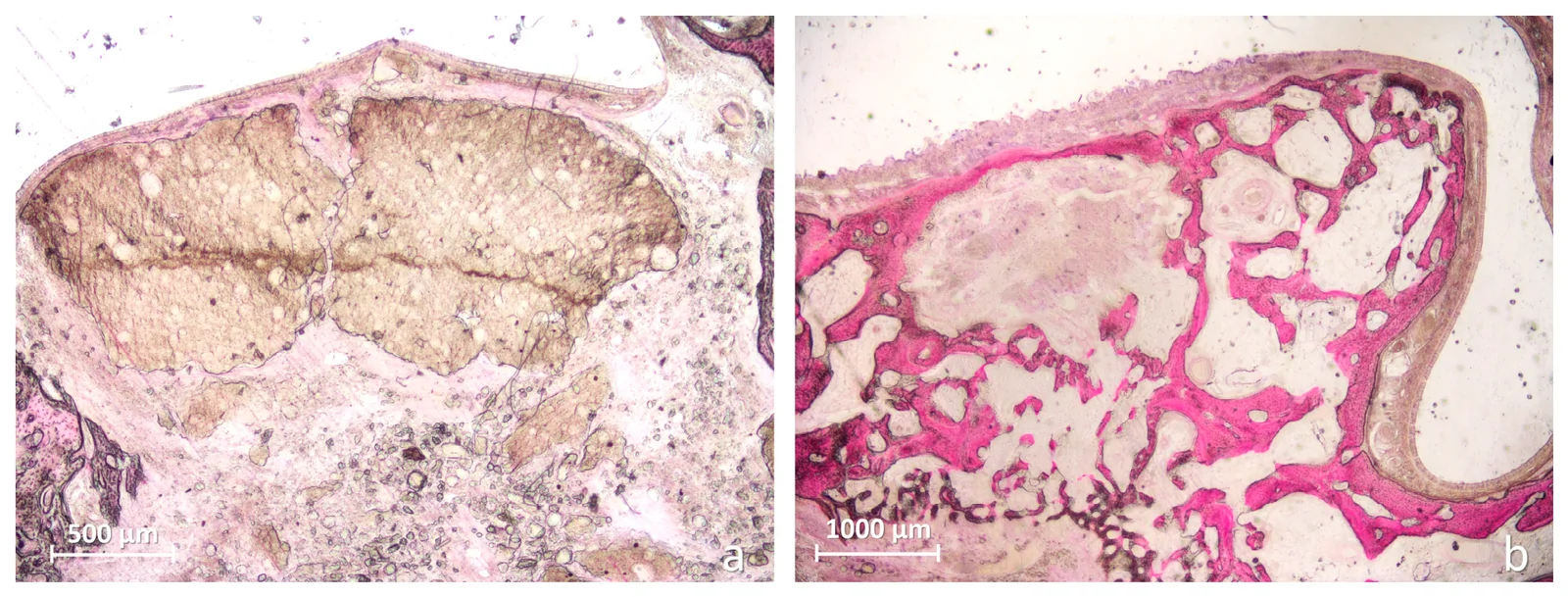
Representative histological images of the ReproBone^®^ novo Paste group after 2 weeks of healing. (**a**) In some specimens, large masses of residual paste-like biomaterial occupied a substantial portion of the elevated compartment and were associated with limited newly formed bone. (**b**) In other specimens, bone formation was more evident, arising mainly in continuity with the native sinus walls and extending toward the central portion of the elevated compartment, while broad areas of residual biomaterial remained present. Acid fuchsin–toluidine blue stain. Scale bars = 500 μm in (**a**), 1000 μm in (**b**).

ReproBone^®^ novo Paste showed marked interspecimen variability. At 2 weeks, some specimens contained large residual biomaterial masses with limited bone formation, whereas others showed more extensive bone growth from the sinus walls (Figure 7).

**Figure 8:**
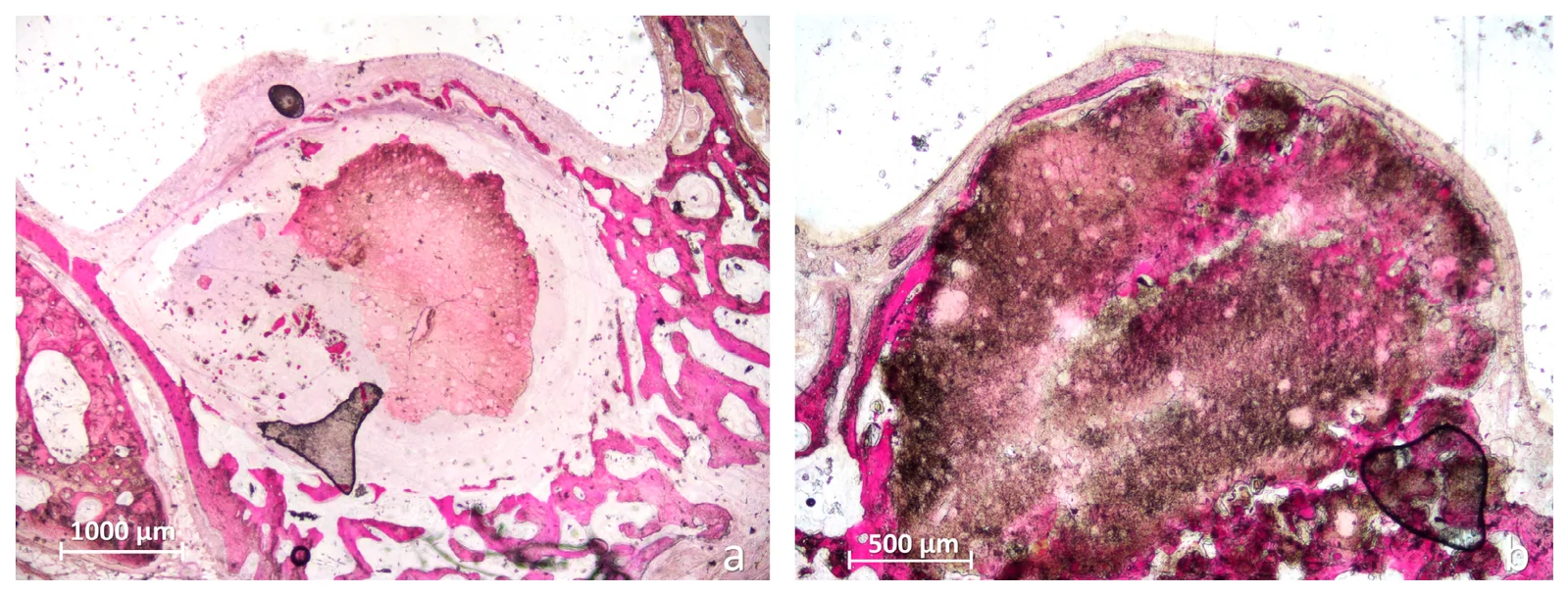
Representative histological images of the ReproBone^®^ novo Paste group after 10 weeks of healing. (**a**) Large residual biomaterial remnants were present in some areas and were surrounded by newly formed bone. (**b**) In other regions, the biomaterial appeared fragmented and disaggregated, with newly formed mineralized tissue closely intermingled with or optically superimposed on residual material, producing an IBN-like pattern. The associated mineralized tissue showed an irregular architecture, and broad soft tissue areas containing fragmented biomaterial were also present. In the sub-Schneiderian region, newly formed bone occasionally showed a more cortical-like organization, whereas the sinus mucosa was generally preserved, with only focal thinning and no perforations. Acid fuchsin-toluidine blue stain. Scale bars = 1000 μm in (**a**), 500 μm in (**b**).

At 10 weeks, residual material ranged from recognizable masses surrounded by bone to fragmented or disaggregated areas containing IBN-like tissue (Figure 8). Mineralized tissue within these areas frequently showed an irregular architecture. The sinus mucosa was generally preserved, with only focal thinning and no perforations.

**Figure 9:**
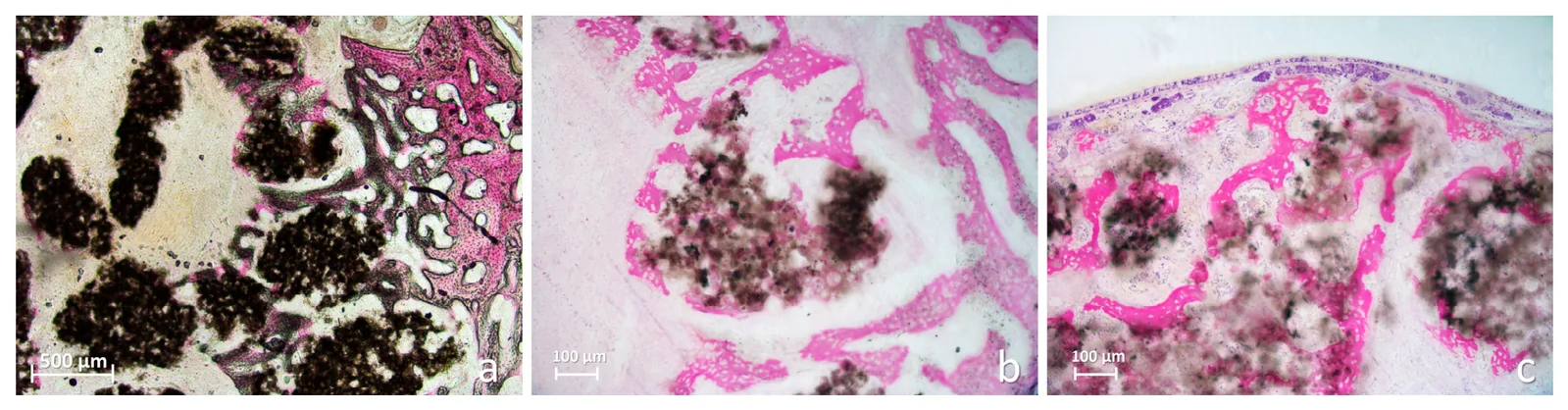
Representative histological images of the Alos Granular group after 2 weeks of healing. (**a**) Newly formed bone extended from the native sinus walls toward the elevated compartment and was closely intermingled with the porous residual biomaterial. Mineralized tissue was also observed within the porous spaces of the material, producing an early IBN-like intragraft pattern. (**b**) Higher-magnification view of the area shown in panel (**a**), with increased transmitted-light intensity to better visualize the close spatial relationship and partial optical overlap between newly formed bone and residual biomaterial. (**c**) Sub-Schneiderian region showing newly formed bone beneath the sinus mucosa, with trabecular structures extending through the grafted compartment. Acid fuchsin-toluidine blue stain. Scale bars = 500 μm in (**a**), 100 μm in (**b**,**c**).

In the Alos Granular group, newly formed bone at 2 weeks extended from the sinus walls and frequently intermingled with the porous residual biomaterial, producing areas with an IBN-like appearance (Figure 9).

**Figure 10:**
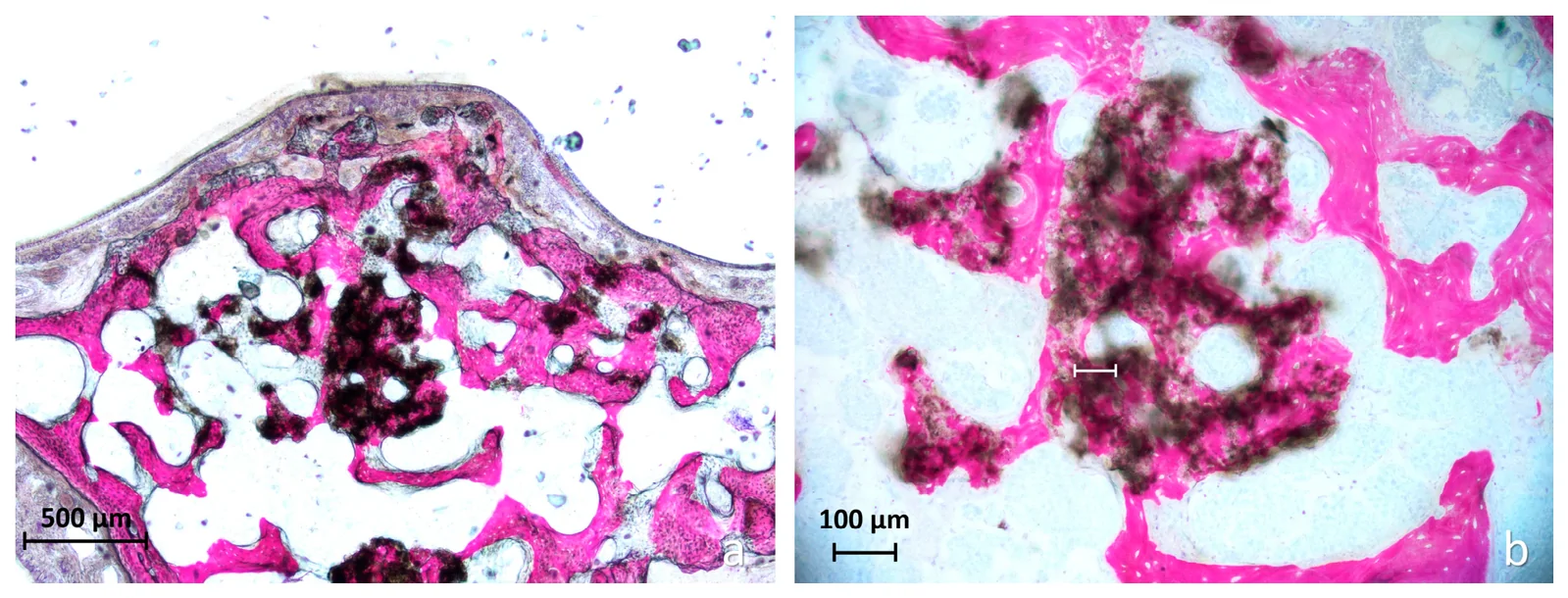
Representative histological images of the Alos Granular group after 10 weeks of healing. (**a**) Newly formed bone was widely distributed throughout the elevated compartment, with a generally regular trabecular architecture; in the sub-Schneiderian region, areas with a more cortical-like organization were also observed. (**b**) Residual biomaterial appeared rarefied and diffusely distributed within the regenerated tissue. Newly formed bone was frequently observed in close spatial association with residual material, including areas suggestive of bone ingrowth within the porous biomaterial remnants. The sinus mucosa was generally preserved, with only scarce focal thinning and no perforations. Acid fuchsin-toluidine blue stain. Scale bars = 500 μm in (**a**), 100 μm in (**b**).

At 10 weeks, bone was widely distributed throughout the elevated compartment, whereas residual material appeared increasingly rarefied and diffusely incorporated within the regenerated tissue (Figure 10). The sinus mucosa was generally preserved, with only focal thinning and no perforations.

### 3.3. Quantitative Histomorphometric Evaluation

**Figure 11:**
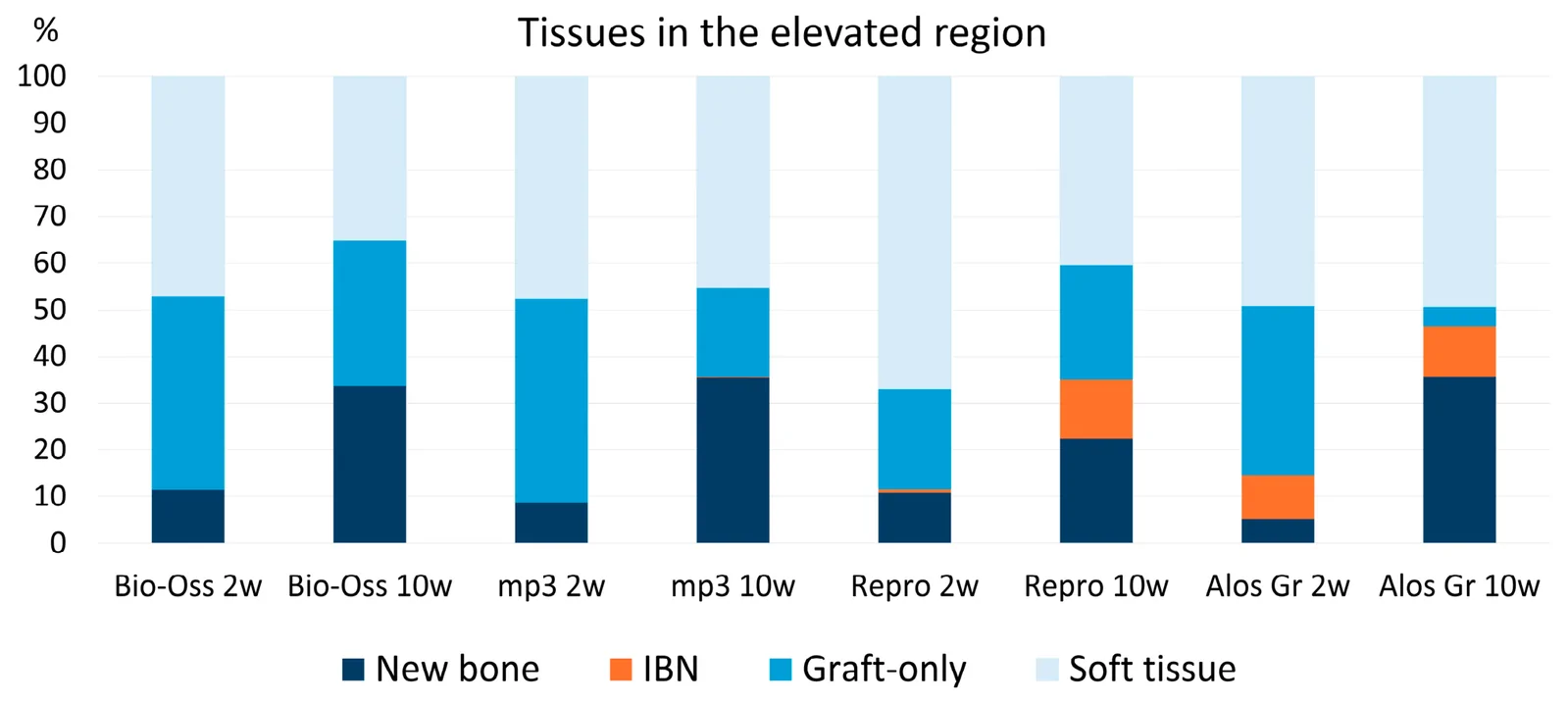
Histomorphometric composition of the elevated sinus compartments after 2 and 10 weeks of healing. Stacked bar charts showing the mean percentages of newly formed bone, IBN-like tissue, graft-only tissue, and soft tissue for each biomaterial. These four categories were mutually exclusive and together represented the total histomorphometric composition of the evaluated compartment. The corresponding standard deviations for each tissue category and biomaterial are reported in Table 1 and Table 2.

Histomorphometric results are summarized in Table 1 and Table 2 and Figure 11.

Residual graft percentage, the primary outcome, did not differ significantly among biomaterials at 2 weeks (Friedman test, *p* = 0.2556). At 10 weeks, a significant difference was detected (*p* = 0.0040), with Bio-Oss^®^ showing a higher residual graft percentage than Alos Granular (adjusted *p* = 0.0024); comparisons with mp3^®^ and ReproBone^®^ novo Paste were not significant.

The combined graft and IBN percentage did not differ significantly among materials at 2 weeks (*p* = 0.2181), whereas a significant overall difference was detected at 10 weeks (*p* = 0.0218). Bio-Oss^®^ showed a higher value than Alos Granular (adjusted *p* = 0.0219), while the comparisons with mp3^®^ and ReproBone^®^ novo Paste were not significant.

Newly formed bone did not differ significantly among biomaterials at either 2 weeks (*p* = 0.1081) or 10 weeks (*p* = 0.0882).

**Figure 12:**
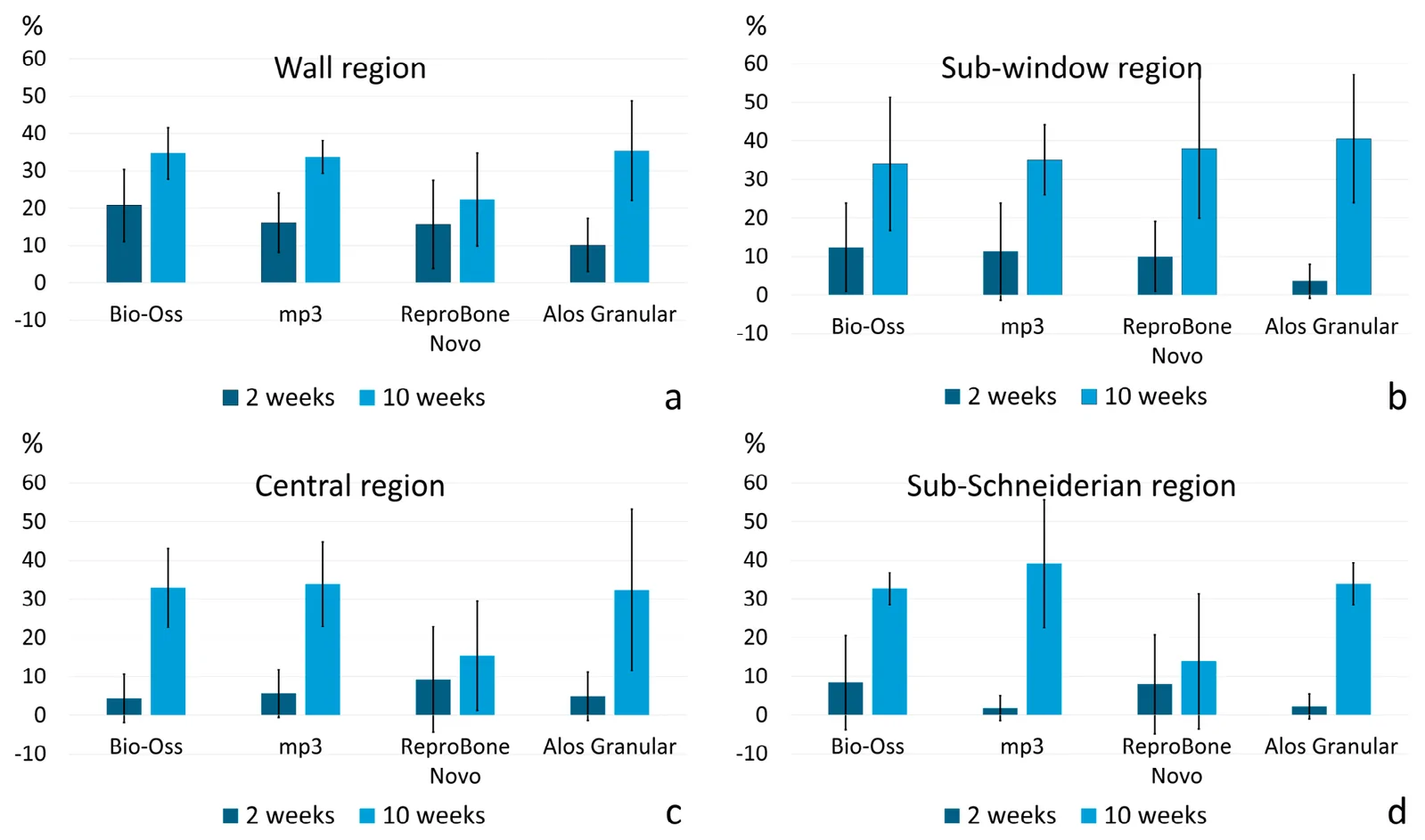
Descriptive regional distribution of newly formed bone within the elevated sinus compartment. Bar charts showing the mean percentages of newly formed bone at the (**a**) wall region (mean value of the medial and lateral walls), (**b**) sub-window region, (**c**) central region, and (**d**) sub-Schneiderian region after 2 and 10 weeks of healing for each biomaterial. Error bars indicate standard deviations. These data are presented descriptively and were not subjected to region-specific inferential statistical analysis. Where the standard deviation exceeded the mean, the lower error bar extended below zero; this does not indicate negative observed percentages.

The descriptive regional analysis showed that bone formation at 2 weeks was predominantly located near the sinus walls and sub-window region, whereas at 10 weeks it was more widely distributed throughout the elevated compartment (Figure 12).

IBN-like tissue did not differ significantly among materials at 2 weeks (*p* = 0.0625). At 10 weeks, a significant overall difference was detected (*p* = 0.0006), with higher percentages in ReproBone^®^ novo Paste and Alos Granular than in Bio-Oss^®^ (adjusted *p* = 0.0219 and *p* = 0.0417, respectively); mp3^®^ did not differ from Bio-Oss^®^. Soft tissue percentages showed no significant differences among biomaterials at either healing interval.

### 3.4. Elevated Compartment Cross-Sectional Area

Cross-sectional area was evaluated descriptively. Compared with the corresponding independent 2-week group means, the 10-week group means were 2.4% lower for Bio-Oss^®^, 19.1% lower for mp3^®^, 6.2% lower for ReproBone^®^ novo Paste, and 55.0% lower for Alos Granular. These values represent descriptive between-group differences and should not be interpreted as longitudinal changes because different animals were evaluated at the two healing intervals.

### 3.5. Descriptive Assessment of Schneiderian Membrane Alterations

**Figure 13:**
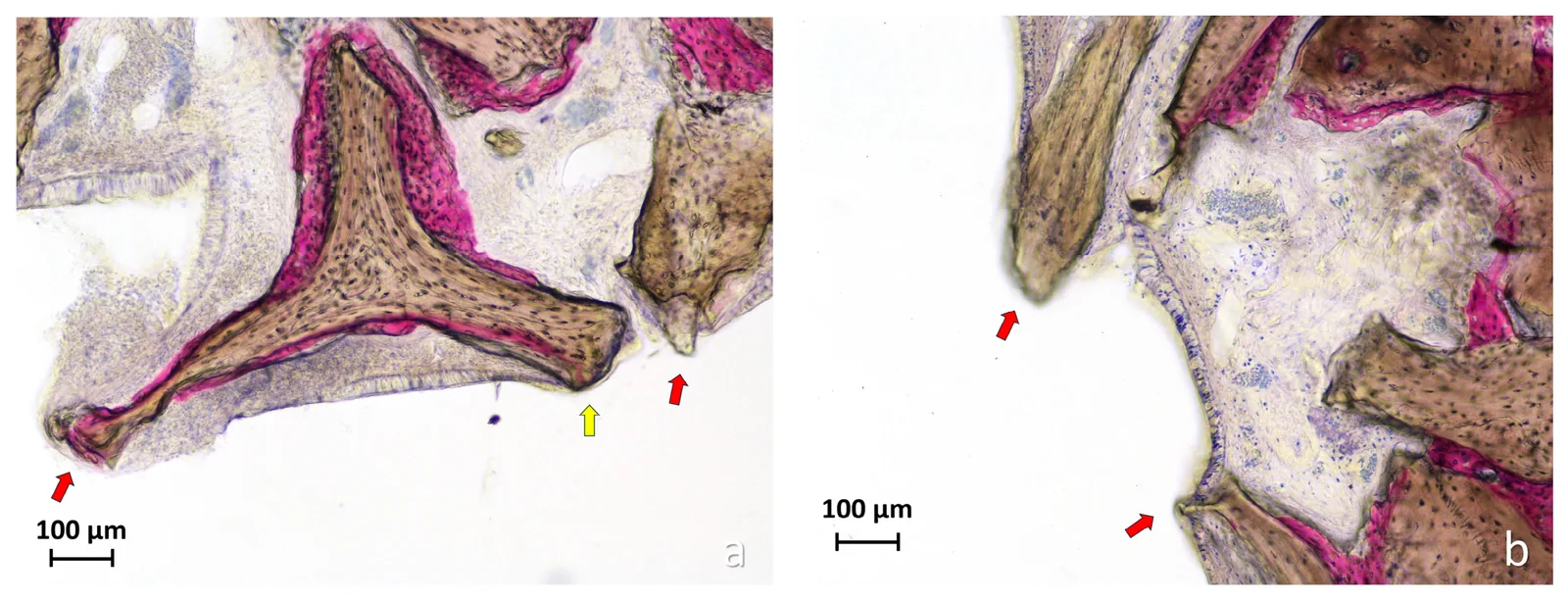
Representative histological images of Schneiderian membrane alterations in the Bio-Oss^®^ group. (**a**) Focal thinning of the sinus mucosa to less than 40 µm is indicated by the yellow arrow. The red arrows indicate mucosal perforations adjacent to Bio-Oss^®^ granules, characterized by interruption of the epithelial lining and loss of the normal structural continuity of the mucosa. At one site, the granule remained covered by a very thin residual layer of connective tissue; however, because epithelial continuity was clearly interrupted, the site was classified as a mucosal perforation according to the predefined histological criterion. (**b**) Additional mucosal perforations, showing complete discontinuity of the epithelial and connective-tissue layers, are indicated by the red arrows. Acid fuchsin-toluidine blue stain. Scale bars = 0.1 mm.

Schneiderian membrane outcomes were evaluated descriptively and were not subjected to inferential comparisons among biomaterials or between healing intervals. Mucosal thinning (<40 μm) was observed in all groups, with the highest absolute number of recorded thinning events at Bio-Oss^®^-treated sites (Table 3; Figure 13). Mucosal perforations were recorded only in the Bio-Oss^®^ group: two perforations in one sinus at 2 weeks and six perforations in four sinuses at 10 weeks. No perforations were recorded at sites treated with mp3^®^, ReproBone^®^ novo Paste, or Alos Granular.

## 4. Discussion

The present study identified distinct healing profiles among the four biomaterials, although no statistically significant differences in newly formed bone percentage were detected. The main differences concerned scaffold persistence and degradation, the spatial relationship between bone and residual biomaterial, the architecture of the regenerated tissue, maintenance of the elevated compartment, and the response of the Schneiderian membrane. These findings support the interpretation of sinus graft healing as a multidimensional process in which newly formed bone percentage alone may not adequately characterize biomaterial performance.

At 10 weeks, the significant difference in residual graft percentage primarily reflected material-specific differences in scaffold persistence and degradation, with Bio-Oss^®^ retaining significantly more recognizable graft than Alos Granular. By contrast, no statistically significant difference was detected in newly formed bone percentage among the materials. Thus, within the investigated sample and healing interval, the more extensive degradation of Alos Granular was not accompanied by a greater proportion of newly formed bone. Conversely, the higher persistence of Bio-Oss^®^ should not be interpreted as evidence of greater bone formation. Moreover, because the histomorphometric outcomes were expressed as percentages, the absence of a significant difference in newly formed bone does not establish equivalence in absolute bone formation or dimensional stability, particularly when the cross-sectional areas of the regenerated compartments differed among materials.

A distinctive histological feature, particularly evident in the synthetic biomaterials, was the occurrence of IBN-like areas. The operational definition used in this study was intended to describe a pattern in which newly formed bone and residual biomaterial were so closely intermingled or optically superimposed that they could not be reliably assigned to conventional bone or graft categories. This should not be interpreted as evidence of a distinct tissue type. Previous observations with calcium phosphate biomaterials have similarly described bone formation within or in close continuity with degrading scaffold structures [40,46].

The higher IBN-like tissue percentages observed with ReproBone^®^ novo Paste and Alos Granular at 10 weeks indicate that intermingling or optical superimposition of newly formed bone and degrading biomaterial occurred more frequently than with the xenogeneic materials. However, a higher IBN-like percentage should not be interpreted as greater bone formation or more favorable biological integration. In ReproBone^®^ novo Paste, these areas were frequently extensive and heterogeneous and were associated with irregularly organized mineralized structures. In contrast, the IBN-like areas observed with Alos Granular occurred in the context of marked scaffold degradation, while the regenerated bone generally retained a recognizable trabecular architecture. Thus, quantitatively similar IBN-like percentages may represent different patterns of scaffold degradation, incorporation, and tissue organization, whose functional significance remains unknown.

From the perspective of subsequent implant placement, residual graft and IBN-like tissue raise two distinct issues. The first concerns residual biomaterial itself. When residual graft is in direct contact with an implant surface, it occupies an area that would otherwise be available for direct bone apposition. In a previous clinical study using Cerabone^®^ (Botiss biomaterials GmbH, Zossen, Germany), residual graft and degraded biomaterial were observed in direct contact with portions of the implant surface [39]. Similarly, in another clinical model using a more progressively remodeling collagenated xenograft, residual biomaterial was present at a limited proportion of the implant interface [47]. Thus, despite the different biomaterials and healing patterns investigated, both studies showed the same fundamental limitation: residual graft reduced the proportion of the implant surface available for direct bone-to-implant contact.

The second issue concerns the structural characteristics of the mineralized tissue associated with degrading biomaterial. In the Cerabone^®^ study, increasing transmitted-light intensity and adjusting the focal plane revealed irregular and poorly trabeculated mineralized structures within, behind, or optically superimposed on degraded graft material [39], producing an appearance comparable to the IBN-like pattern observed in the present study. This observation raises a different question from that posed by residual graft alone: even when mineralized tissue is present, its architecture and maturation may differ from those of conventionally organized trabecular bone. Although the available evidence does not demonstrate that such IBN-like structures impair osseointegration, it suggests that their biological relevance may depend not only on their amount, but also on the structural quality and organization of the associated mineralized tissue [39,40].

Bio-Oss^®^ showed the conventional surface-guided osteoconductive pattern expected for a slowly resorbing DBBM, with residual particles providing a persistent scaffold for deposition and connection of newly formed trabeculae [48,49]. A broadly comparable trabecular pattern was observed with mp3^®^, consistent with previous histological observations of collagenated porcine xenografts [50,51,52,53]. The focal IBN-like component identified in one mp3^®^ specimen did not appear to alter the predominantly surface-related organization of the regenerated bone. This observation also indicates that limited IBN-like features are not necessarily restricted to synthetic biomaterials or associated with disruption of trabecular architecture.

Alos Granular exhibited a different balance between scaffold degradation and tissue replacement. Its extensive degradation was associated with limited persistence of recognizable residual biomaterial, while the regenerated bone generally maintained a recognizable trabecular organization despite the presence of IBN-like areas. Thus, IBN-like tissue was not invariably associated with structurally atypical bone. However, the marked reduction in cross-sectional area observed between the independent healing groups suggests that rapid scaffold degradation was not fully compensated by maintenance of the elevated compartment.

ReproBone^®^ novo Paste showed a contrasting pattern. Residual biomaterial and IBN-like areas were frequently extensive and heterogeneous, and the associated mineralized structures often lacked the clearly recognizable trabecular architecture observed with the xenogeneic materials and, to a greater extent, with Alos Granular. This finding does not demonstrate inferior bone formation or mechanical competence, neither of which was directly tested. It does, however, emphasize that similar percentages of newly formed bone may coexist with markedly different structural organization of the regenerated compartment. The relatively preserved mean cross-sectional area should also be interpreted cautiously because the material frequently appeared disaggregated and diffusely distributed rather than maintaining its original graft architecture.

Cross-sectional area was included as a descriptive indicator of compartment maintenance and not as a volumetric measurement. Differences were already present between materials at the early healing interval and may reflect differences in consistency, particle structure, hydration, packing, spreading beneath the sinus mucosa, early degradation, and sectioning plane. Consequently, differences between the independent 2- and 10-week groups cannot be interpreted as longitudinal dimensional changes. Nevertheless, the markedly reduced area associated with Alos Granular illustrates that scaffold degradation, residual biomaterial persistence, and maintenance of the augmented compartment represent related but distinct aspects of graft behavior.

The Schneiderian membrane findings further highlight the importance of evaluating biomaterial–tissue interactions beyond bone formation. Localized mucosal thinning occurred with all materials, whereas perforations were observed only adjacent to Bio-Oss^®^ particles. Previous experimental studies have also shown that persistent graft particles may induce thinning or structural damage to the sinus mucosa [41,42]. The present observations are compatible with the possibility that prolonged contact with rigid, slowly resorbing particles contributes to localized mucosal injury during healing. However, an unrecognized intraoperative membrane injury cannot be completely excluded, and the use of different animals at the two healing intervals prevents interpretation of the findings as direct evidence of progressive damage over time. Schneiderian membrane perforation remains clinically relevant because of its potential implications for sinus augmentation procedures and postoperative complications [54,55,56,57,58,59]. However, the outcomes reported in the present study were evaluated descriptively, and no inferential comparisons among biomaterials were performed. Consequently, the observed distribution of mucosal thinning and perforations should not be interpreted as evidence of a statistically supported material effect.

Taken together, the findings indicate that none of the investigated materials can be characterized as uniformly superior on the basis of the present outcomes. Bio-Oss^®^ and mp3^®^ provided persistent scaffolds associated with conventional surface-related osteoconduction; Alos Granular showed extensive degradation with preservation of generally recognizable trabecular bone but reduced maintenance of the elevated compartment; and ReproBone^®^ novo Paste showed greater structural heterogeneity, with extensive graft–bone intermingling and irregular mineralized architecture. These patterns illustrate the need to consider scaffold persistence, mode of incorporation, tissue architecture, space maintenance, and mucosal response together rather than relying on newly formed bone percentage alone.

Several limitations should be acknowledged. The rabbit sinus model allows standardized evaluation of early healing but does not fully reproduce the anatomical, dimensional, and functional conditions of the human maxillary sinus [60]. The number of animals was limited, which may have reduced the statistical power of some comparisons, particularly for outcomes characterized by substantial inter-animal variability. Secondary and exploratory findings should therefore be interpreted cautiously. Different animals were evaluated at 2 and 10 weeks, preventing paired longitudinal assessment. One female replacement animal was included among otherwise male animals; although all biomaterials were evaluated within each rabbit, sex-related effects could not be specifically assessed.

The study did not include either an ungrafted elevated compartment or an autogenous bone group. Although Bio-Oss^®^ was used as a clinically established reference material, it does not represent either a negative control or an autogenous biological benchmark. Consequently, the findings allow relative comparisons among the four biomaterials but do not establish their performance in relation to spontaneous healing of an ungrafted compartment or to autogenous bone.

In addition, implants were not placed in the regenerated compartments, and the functional competence of IBN-like mineralized tissue and its potential influence on osseointegration remain unknown. Finally, follow-up was limited to 10 weeks, and longer-term studies are required to determine whether these graft–bone patterns undergo further maturation, replacement, or persistence.

## 5. Conclusions

Within the limitations of this exploratory study:

- Newly formed bone percentages did not differ significantly among the four biomaterials at either 2 or 10 weeks.
- At 10 weeks, Bio-Oss^®^ showed significantly higher residual graft and combined graft and IBN percentages than Alos Granular, consistent with greater scaffold persistence. At the same healing interval, IBN-like tissue percentages were higher in ReproBone^®^ novo Paste and Alos Granular than in Bio-Oss^®^.
- Bio-Oss^®^ and mp3^®^ predominantly showed conventional surface-related osteoconduction within a recognizable trabecular network. Alos Granular showed extensive scaffold degradation and limited residual graft persistence, although the regenerated bone generally maintained a recognizable trabecular organization. ReproBone^®^ novo Paste showed heterogeneous graft–bone intermingling, with extensive graft and IBN areas frequently associated with irregular and poorly trabeculated mineralized tissue.
- Overall, the findings indicate that the biological performance of sinus grafting materials cannot be characterized by newly formed bone percentage alone. Residual graft persistence, degradation pattern, graft–bone integration, tissue architecture, maintenance of the elevated compartment, and Schneiderian membrane response should also be considered. Further studies including implant placement and direct assessment of the bone-to-implant interface are required to determine the functional relevance of persistent IBN-like graft–bone complexes.

## Figures and Tables

**Table 1:** Histomorphometric composition of the elevated sinus compartments after 2 weeks of healing. Values are presented as mean ± standard deviation (%). New bone, graft-only tissue, IBN-like tissue, and soft tissue are mutually exclusive categories and sum to 100%. Combined graft and IBN is a derived outcome calculated as the sum of graft-only and IBN-like tissue percentages and is not included in that total.

BioMaterial	New Bone	Graft-Only Tissue	IBN-Like Tissue	Soft Tissue	Combined Graft and IBN
Bio-Oss	11.5 ± 6.0	41.4 ± 8.7	0.0 ± 0.0	47.1 ± 5.6	41.4 ± 8.7
mp3	8.7 ± 4.4	43.7 ± 9.2	0.0 ± 0.0	47.6 ± 9.1	43.7 ± 9.2
ReproBone	10.8 ± 8.3	21.3 ± 26.8	0.9 ± 2.1	67.1 ± 24.3	22.2 ± 28.4
Alos	5.2 ± 2.5	36.2 ± 17.1	9.4 ± 11.7	49.3 ± 26.8	45.6 ± 25.1

**Table 2:** Histomorphometric composition of the elevated sinus compartments after 10 weeks of healing. Values are presented as mean ± standard deviation (%). New bone, graft-only tissue, IBN-like tissue, and soft tissue are mutually exclusive categories and sum to 100%. Combined graft and IBN is a derived outcome calculated as the sum of graft-only and IBN-like tissue percentages and is not included in that total.

BioMaterial	New Bone	Graft-Only Tissue	IBN-like Tissue	Soft Tissue	Combined Graft and IBN
Bio-Oss	33.6 ± 7.1	31.4 ± 6.0	0.0 ± 0.0	35.1 ± 8.0	31.4 ± 6.0
mp3	35.4 ± 5.7	19.2 ± 8.8	0.2 ± 0.5	45.2 ± 12.1	19.4 ± 9.1
ReproBone	22.4 ± 10.9	24.5 ± 24.3	12.6 ± 10.9	40.4 ± 22.2	37.2 ± 30.0
Alos	35.6 ± 11.2	4.2 ± 3.6	10.8 ± 4.9	49.4 ± 13.4	15.0 ± 6.3

**Table 3:** Descriptive assessment of Schneiderian membrane alterations after 2 and 10 weeks of healing. Mucosal thinning was defined as a mucosal thickness <40 µm. Thickness values at thinned sites are presented as mean ± standard deviation. All outcomes are presented descriptively; no inferential comparisons among biomaterials or between healing intervals were performed.

		Thinned Mucosal Sites		Perforations (n)	
		Mean Thickness (µm)	Events (n)	Events	Sinuses with ≥1 Perforation
Bio-Oss	2 weeks	25 ± 3	18	2	1
	10 weeks	26 ± 9	19	6	4
mp3	2 weeks	28 ± 4	9	0	0
	10 weeks	20 ± 10	6	0	0
ReproBone Novo	2 weeks	31 ± 3	7	0	0
	10 weeks	17 ± 6	3	0	0
Alos Granular	2 weeks	20 ± 6	13	0	0
	10 weeks	22 ± 11	3	0	0

## Data Availability

The data supporting the findings of this study are available from the corresponding author upon reasonable request.

## References

[1] Sabri H., Saleh M.H.A., Nava P., Scaini R., Testori T., Del Fabbro M. (2026). Long-term outcomes of lateral sinus floor elevation: A machine-learning analysis, systematic review, and meta-analysis of predictive factors. Periodontol. 2000.

[2] Sabri H., Saleh M.H.A., Zimmer J.M., Schwarz F., Wang H.L. (2026). Patient-Reported Outcome Measures (PROMs) and Clinician-Reported Outcomes (ClinROs) in Sinus Floor Elevation for Implant Rehab in the Edentulous Maxilla: A Systematic Review and COSMIN Analysis. Clin. Oral Implant. Res..

[3] Ghodsian D., D’Jesús S., Sánchez-Labrador L., Cobo-Vázquez C.M., Cortés-Bretón Brinkmann J., Martínez-González J.M., Meniz-García C. (2024). Maxillary Sinus Augmentation with Autogenous Tooth Grafting Material: A Systematic Review. Biomimetics.

[4] Busenlechner D., Huber C.D., Vasak C., Dobsak A., Gruber R., Watzek G. (2009). Sinus augmentation analysis revised: The gradient of graft consolidation. Clin. Oral Implant. Res..

[5] Lambert F., Léonard A., Drion P., Sourice S., Layrolle P., Rompen E. (2011). Influence of space-filling materials in subantral bone augmentation: Blood clot vs. autogenous bone chips vs. bovine hydroxyapatite. Clin. Oral Implant. Res..

[6] Xu H., Shimizu Y., Asai S., Ooya K. (2004). Grafting of deproteinized bone particles inhibits bone resorption after maxillary sinus floor elevation. Clin. Oral Implant. Res..

[7] Corbella S., Taschieri S., Weinstein R., Del Fabbro M. (2016). Histomorphometric outcomes after lateral sinus floor elevation procedure: A systematic review of the literature and meta-analysis. Clin. Oral Implant Res..

[8] Zhang L., Si M., Shi J., Yang G., Shi Y. (2019). Evaluation of three-dimensional contraction of the volume of grafts after staged augmentation of the sinus floor. Br. J. Oral Maxillofac. Surg..

[9] Temmerman A., Van Dessel J., Cortellini S., Jacobs R., Teughels W., Quirynen M. (2017). Volumetric changes of grafted volumes and the Schneiderian membrane after transcrestal and lateral sinus floor elevation procedures. J. Clin. Periodontol..

[10] Jensen T., Schou S., Svendsen P.A., Forman J.L., Gundersen H.J.G., Terheyden H., Holmstrup P. (2012). Volumetric changes of the graft after maxillary sinus floor augmentation with Bio-Oss and autogenous bone in different ratios: A radiographic study in minipigs. Clin. Oral Implant. Res..

[11] Canellas J.V.D.S., Drugos L., Ritto F.G., Fischer R.G., Medeiros P.J.D. (2021). Xenograft materials in maxillary sinus floor elevation surgery: A systematic review with network meta-analyses. Br. J. Oral Maxillofac. Surg..

[12] Li X., Lin S.C., Duan S.Y. (2023). The impact of deproteinized bovine bone particle size on histological outcomes in sinus floor elevation: A systematic review and meta-analysis. Int. J. Implant Dent..

[13] Li Y., Zhou W., Li P., Luo Q., Li A., Zhang X. (2022). Comparison of the osteogenic effectiveness of an autogenous demineralised dentin matrix and Bio-Oss^®^ in bone augmentation: A systematic review and meta-analysis. Br. J. Oral Maxillofac. Surg..

[14] Figueiredo M., Henriques J., Martins G., Guerra F., Judas F., Figueiredo H. (2010). Physicochemical characterization of biomaterials commonly used in dentistry as bone substitutes—Comparison with human bone. J. Biomed. Mater. Res. B Appl. Biomater..

[15] Schmitt C.M., Wiesheu S., Schlegel K.A., Adler W., Kesting M.R., Matta R.E., Möst T. (2024). Three-dimensional assessment after maxillary sinus grafting with a bovine, a porcine, and a synthetic bone substitute material. A randomized controlled clinical trial. Int. J. Comput. Dent..

[16] Zamure-Damberga L., Radzins O., Salms G., Zolovs M., Bokvalde Z., Neimane L. (2024). Long-Term Volumetric Stability of Maxillary Sinus Floor Augmentation Using a Xenograft Bone Substitute and Its Combination with Autologous Bone: A 6+ Year Retrospective Follow-Up Study Using Cone Beam Computed Tomography. Dent. J..

[17] Piattelli M., Favero G.A., Scarano A., Orsini G., Piattelli A. (1999). Bone reactions to anorganic bovine bone (Bio-Oss) used in sinus augmentation procedures: A histologic long-term report of 20 cases in humans. Int. J. Oral Maxillofac. Implant..

[18] Valentini P., Bosshardt D.D. (2018). 20-Year Follow-up in Maxillary Sinus Floor Elevation Using Bovine-Derived Bone Mineral: A Case Report with Histologic and Histomorphometric Evaluation. Int. J. Oral Maxillofac. Implant..

[19] Xu H., Shimizu Y., Onodera K., Ooya K. (2005). Long-term outcome of augmentation of the maxillary sinus using deproteinised bone particles experimental study in rabbits. Br. J. Oral Maxillofac. Surg..

[20] Barone A., Ricci M., Grassi R.F., Nannmark U., Quaranta A., Covani U. (2013). A 6-month histological analysis on maxillary sinus augmentation with and without use of collagen membranes over the osteotomy window: Randomized clinical trial. Clin. Oral Implant. Res..

[21] Correia F., Pozza D.H., Gouveia S., Felino A.C., Faria-Almeida R. (2021). Advantages of Porcine Xenograft over Autograft in Sinus Lift: A Randomised Clinical Trial. Materials.

[22] Pistilli R., Canullo L., Pesce P., Pistilli V., Caponio V.C.A., Sbricoli L. (2022). Guided implant surgery and sinus lift in severely resorbed maxillae: A retrospective clinical study with up to 10 years of follow-up. J. Dent..

[23] Taniguchi Z., Esposito M., Xavier S.P., Silva E.R., Botticelli D., Buti J., Baba S. (2025). On the Use of a Sticky Bone Substitute in the Presence of a Ruptured Sinus Membrane During Sinus Elevation Procedures: An Experimental Rabbit Study. Int. J. Oral Maxillofac. Implant..

[24] Wenz B., Oesch B., Horst M. (2001). Analysis of the risk of transmitting bovine spongiform encephalopathy through bone grafts derived from bovine bone. Biomaterials.

[25] Bucchi C., Del Fabbro M., Arias A., Fuentes R., Mendes J.M., Ordonneau M., Orti V., Manzanares-Céspedes M.C. (2019). Multicenter study of patients’ preferences and concerns regarding the origin of bone grafts utilized in dentistry. Patient Prefer Adherence.

[26] Eliaz N., Metoki N. (2017). Calcium phosphate bioceramics: A review of their history, structure, properties, coating technologies and biomedical applications. Materials.

[27] Bozza B., Pesce P., Baldi D., Bagnasco F., Migliorati M., De Angelis N. (2025). Synthetic Biomaterials for Alveolar Bone Regeneration: A Systematic Review of Clinical Evidence. Materials.

[28] Ivanova N., Ivanov S., Peev S., Dikova T. (2025). Types of Bone Substitutes and Their Application in Regenerative Medicine: A Systematic Review. J. Funct. Biomater..

[29] Somngam C., Samartkit S., Kanchanasurakit S., Strietzel F.P., Khongkhunthian P. (2025). New bone formation of biphasic calcium phosphate bone substitute material: A systematic review and network meta-analysis of randomized controlled trials (RCTs). Int. J. Implant Dent..

[30] Kerckhofs G., Chai Y.C., Luyten F.P., Geris L. (2016). Combining microCT-based characterization with empirical modelling as a robust screening approach for the design of optimized CaP-containing scaffolds for progenitor cell-mediated bone formation. Acta Biomater..

[31] Chai Y.C., Roberts S.J., Desmet E., Kerckhofs G., van Gastel N., Geris L., Carmeliet G., Schrooten J., Luyten F.P. (2012). Mechanisms of ectopic bone formation by human osteoprogenitor cells on CaP biomaterial carriers. Biomaterials.

[32] Roberts S.J., Geris L., Kerckhofs G., Desmet E., Schrooten J., Luyten F.P. (2011). The combined bone forming capacity of human periosteal derived cells and calcium phosphates. Biomaterials.

[33] Rezwan K., Chen Q.Z., Blaker J.J., Boccaccini A.R. (2006). Biodegradable and bioactive porous polymer/inorganic composite scaffolds for bone tissue engineering. Biomaterials.

[34] Ceccarelli G., Presta R., Lupi S.M., Giarratana N., Bloise N., Benedetti L., Cusella De Angelis M.G., Rodriguez YBaena R. (2017). Evaluation of Poly(Lactic-co-glycolic) Acid Alone or in Combination with Hydroxyapatite on Human-Periosteal Cells Bone Differentiation and in Sinus Lift Treatment. Molecules.

[35] Félix Lanao R.P., Jonker A.M., Wolke J.G., Jansen J.A., van Hest J.C., Leeuwenburgh S.C. (2013). Physicochemical properties and applications of poly(lactic-co-glycolic acid) for use in bone regeneration. Tissue Eng. Part B Rev..

[36] Gentile P., Chiono V., Carmagnola I., Hatton P.V. (2014). An overview of poly(lactic-co-glycolic) acid (PLGA)-based biomaterials for bone tissue engineering. Int. J. Mol. Sci..

[37] Polimeni G., Koo K.-T., Pringle G.A., Agelan A., Safadi F.F., Wikesjö U.M.E. (2008). Histopathological observations of a polylactic acid-based device intended for guided bone/tissue regeneration. Clin. Implant Dent. Relat. Res..

[38] Herranz-Diez C., Crawford A., Goodchild R.L., Hatton P.V., Miller C.A. (2022). Stimulation of Metabolic Activity and Cell Differentiation in Osteoblastic and Human Mesenchymal Stem Cells by a Nanohydroxyapatite Paste Bone Graft Substitute. Materials.

[39] Kotsu M., Apaza Alccayhuaman K.A., Ferri M., Iezzi G., Piattelli A., Fortich Mesa N., Botticelli D. (2022). Osseointegration at Implants Installed in Composite Bone: A Randomized Clinical Trial on Sinus Floor Elevation. J. Funct. Biomater..

[40] Tanaka K., Botticelli D., Canullo L., Baba S., Xavier S.P. (2020). New bone ingrowth into β-TCP/HA graft activated with argon plasma: A histomorphometric study on sinus lifting in rabbits. Int. J. Implant Dent..

[41] Omori Y., Botticelli D., Migani S., Balan V.F., Pires Godoy E., Xavier S.P. (2022). Sinus mucosal damage triggered by synthetic or xenogeneic bone substitutes: A histological analysis in rabbits. J. Funct. Biomater..

[42] Nakajima Y., Botticelli D., De Rossi E.F., Ferreira Balan V., Pires Godoy E., Ricardo Silva E., Xavier S.P. (2023). Schneiderian membrane collateral damage caused by collagenated and non-collagenated xenografts: A histological study in rabbits. Dent. J..

[43] Percie du Sert N., Hurst V., Ahluwalia A., Alam S., Avey M.T., Baker M., Browne W.J., Clark A., Cuthill I.C., Dirnagl U. (2020). The ARRIVE guidelines 2.0: Updated guidelines for reporting animal research. Br. J. Pharmacol..

[44] Maniwa N., Xavier S.P., Scombatti de Souza S.L., Silva E.R., Botticelli D., Morinaga K., Baba S. (2024). Sequential Bone Repair in Rabbit Sinus Lifts Using Bio-Oss and Hyaluronic Acid-Polynucleotide Gel (Regenfast). J. Funct. Biomater..

[45] Faul F., Erdfelder E., Buchner A., Lang A.G. (2009). Statistical power analyses using G*Power 3.1: Tests for correlation and regression analyses. Behav. Res. Methods.

[46] Mangano C., Perrotti V., Shibli J.A., Mangano F., Ricci L., Piattelli A., Iezzi G. (2013). Maxillary sinus grafting with biphasic calcium phosphate ceramics: Clinical and histologic evaluation in man. Int. J. Oral Maxillofac. Implant..

[47] Morimoto A., Kobayashi N., Ferri M., Iezzi G., Piattelli A., Fortich Mesa N., Botticelli D. (2022). Influence on Implant Bone Healing of a Collagen Membrane Placed Subjacent the Sinus Mucosa-A Randomized Clinical Trial on Sinus Floor Elevation. Dent. J..

[48] Busenlechner D., Tangl S., Mair B., Fugger G., Gruber R., Redl H., Watzek G. (2008). Simultaneous in vivo comparison of bone substitutes in a guided bone regeneration model. Biomaterials.

[49] Korzinskas T., Schnettler R., Rimashevskiy D., Malik B., Dakenov B., Bagdoniene D., Jung O., Barbeck M. (2026). Low-temperature Versus High-temperature Sintering: Regenerative and Osteoimmunological Insights from Bio-Oss^®^, Ti-Oss^®^ in Sinus Lift Surgery. In Vivo.

[50] Barone A., Ricci M., Covani U., Nannmark U., Azarmehr I., Calvo-Guirado J.L. (2012). Maxillary sinus augmentation using prehydrated corticocancellous porcine bone: Hystomorphometric evaluation after 6 months. Clin. Implant Dent. Relat. Res..

[51] Giuliani A., Iezzi G., Mazzoni S., Piattelli A., Perrotti V., Barone A. (2018). Regenerative properties of collagenated porcine bone grafts in human maxilla: Demonstrative study of the kinetics by synchrotron radiation microtomography and light microscopy. Clin. Oral Investig..

[52] Falacho R.I., Palma P.J., Marques J.A., Figueiredo M.H., Caramelo F., Dias I., Viegas C., Guerra F. (2021). Collagenated Porcine Heterologous Bone Grafts: Histomorphometric Evaluation of Bone Formation Using Different Physical Forms in a Rabbit Cancellous Bone Model. Molecules.

[53] Otero-Cruz A.R., Pecci-Lloret M.P., Pérez-Guzmán N., El-Akrout A.E., Ruiz-Roca J.A. (2026). Autologous Bone Versus Xenograft and Their Combination in Vertical Ridge Augmentation: An Analysis of Graft Resorption and Implant Survival-A Systematic Review. Dent. J..

[54] Díaz-Olivares L.A., Cortés-Bretón Brinkmann J., Martínez-Rodríguez N., Martínez-González J.M., López-Quiles J., Leco-Berrocal I., Meniz-García C. (2021). Management of Schneiderian membrane perforations during maxillary sinus floor augmentation with lateral approach in relation to subsequent implant survival rates: A systematic review and meta-analysis. Int. J. Implant Dent..

[55] Becker S.T., Terheyden H., Steinriede A., Behrens E., Springer I., Wiltfang J. (2008). Prospective observation of 41 perforations of the Schneiderian membrane during sinus floor elevation. Clin. Oral Implant. Res..

[56] Ye M., Lin X., Liu W., Calatrava J., Huang W., Wang H.L. (2025). Residual ridge height as a potential risk factor for membrane perforation during lateral-window sinus elevation surgery: A systematic review and meta-analysis. BMC Oral Health.

[57] Urban I.A., Nagursky H., Church C., Lozada J.L. (2012). Incidence, diagnosis, and treatment of sinus graft infection after sinus floor elevation: A clinical study. Int. J. Oral Maxillofac. Implant..

[58] Park W.B., Han J.Y., Kang P., Momen-Heravi F. (2019). The clinical and radiographic outcomes of Schneiderian membrane perforation without repair in sinus elevation surgery. Clin. Implant Dent. Relat. Res..

[59] Doud Galli S.K., Lebowitz R.A., Giacchi R.J., Glickman R., Jacobs J.B. (2001). Chronic sinusitis complicating sinus lift surgery. Am. J. Rhinol..

[60] Casteleyn C., Cornillie P., Hermens A., Van Loo D., Van Hoorebeke L., van den Broeck W., Simoens P. (2010). Topography of the rabbit paranasal sinuses as a prerequisite to model human sinusitis. Rhinology.

